# Protein intake adequacy among Nigerian infants, children, adolescents and women and protein quality of commonly consumed foods

**DOI:** 10.1017/S0954422419000222

**Published:** 2020-06

**Authors:** Judith de Vries-ten Have, Adedotun Owolabi, Jan Steijns, Urszula Kudla, Alida Melse-Boonstra

**Affiliations:** 1 Division of Human Nutrition and Health, Wageningen University and Research, PO Box 9101, 6700 HB Wageningen, The Netherlands; 2 FrieslandCampina WAMCO, Lagos, Nigeria; 3 FrieslandCampina, 3818 LE Amersfoort, The Netherlands

**Keywords:** Nigeria, Protein intake, Essential amino acids, Digestible indispensable amino acid score (DIAAS), Diet

## Abstract

Protein is important for growth, maintenance and protection of the body. Both adequacy of protein quantity and protein quality in the diet are important to guarantee obtaining all the essential amino acids. Protein–energy malnutrition is widely present in developing countries such as Nigeria and might result in stunting and wasting. Needs for protein differ depending on age and physiological status and are higher during growth, pregnancy and lactation. The present review assessed protein quantity and quality in diets of Nigerian infants, children, adolescents, and pregnant and lactating women. Literature reviews and calculations were performed to assess adequacy of Nigerian protein intake and to examine the Nigerian diet. The digestible indispensable amino acid score was used to calculate protein quality of nine Nigerian staple foods and of a mixture of foods. The Nigerian population had mostly adequate protein intake when compared with the most recent protein recommendations by the FAO (2013) and WHO/FAO/UNU (2007). An important exception was the protein intake of adolescent girls and pregnant and lactating women. Most of the assessed Nigerian plant-based staple foods were of low protein quality and predominantly lacked the amino acid lysine. The addition of animal-source foods can bridge the protein quality gap created by predominance of plant-based foods in the Nigerian diet. The methodology of this review can be applied to other low- and middle-income countries where diets are often plant-based and lack variety, which might influence protein intake adequacy.

## Introduction

Proteins, made up of amino acids, are essential elements in a diet. They are used for growth (i.e. building tissues and fluids) and replacement of lost amino acids and thus maintenance of approximately 25 000 proteins coded by the human genome^(1,2)^. Furthermore, they have regulatory and catalytic functions, protect against infections and can serve as a source of energy^(1,2)^. Nine essential amino acids cannot be synthesised by the body and should, therefore, be obtained from the diet. The essential amino acids are: histidine (His), isoleucine (Ileu), leucine (Leu), lysine (Lys), methionine (Met), phenylalanine (Phe), threonine (Thr), tryptophan (Tryp) and valine (Val). Conditionally essential amino acids cysteine (Cys) and tyrosine (Tyr) are often combined respectively with Met as sulfur amino acids (SAA) and Phe as aromatic amino acids (AAA) for the purpose of calculating dietary requirements.

Adequate protein intake is especially important for infants, children and adolescents since these life stages are characterised by rapid increases in height, weight, development and function maturation, which require higher protein intake^(2)^. Furthermore, pre-pregnancy underweight has been shown to be associated with low birth weight^(2,3)^. Pregnant and lactating women also have increased protein intake demands for net tissue deposit or milk formation^(2)^. The period from conception until the age of 2 years is especially important for physical, mental and cognitive growth, development and health of the infant^(3)^. However, in the case of developing countries including Nigeria, this period is often characterised by protein–energy malnutrition (PEM), which interferes with optimal growth and development^(3)^.

PEM, a form of undernutrition, indicates a lack of supply to the body or underutilisation of protein and energy^(4,5)^. In 2012, PEM was the tenth leading cause of death in the Nigerian population, accounting for 2·5 % of total deaths^(6)^. PEM can also result in wasting, i.e. acute malnutrition, and stunting, i.e. chronic malnutrition, affecting, respectively, 7 % (and 2 % severely wasted) and 37 % (and 19 % severely stunted) of children in Nigeria under the age of 5 years^(3)^. Although wasting has decreased in the recent years (from 18 % in 2013 to 7 % in 2018)^(7,8)^, there has been no improvement in stunting (in 2013 stunting was 37 %)^(7)^. Additional protein intake might be beneficial for catch-up growth in children who are stunted and for rapid weight gain in children who are wasted^(2)^.

PEM may be further exacerbated by the fact that many children in Nigeria carry the additional burden of different infections. In Nigeria, acute respiratory infections, diarrhoea, sepsis, HIV and AIDS are among the most prevalent causes of total deaths in children under the age of 5 years, with, respectively, 15, 10, 5 and 3 % of deaths^(6)^. For the total population, lower respiratory diseases and HIV/AIDS are the top two leading causes of death accounting for, respectively, 13·9 and 10·4 % of deaths^(6)^. Individuals who suffer from infections, such as HIV/AIDS, tuberculosis, acute diarrhoea, acute respiratory infections and sepsis, activate new metabolic pathways that utilise amino acids, and might, therefore, benefit from higher protein intake to replace specific amino acids^(2,9)^.

In Nigeria, low-cost foods rich in good-quality protein are scant^(10)^, which makes it difficult to meet protein and amino acid requirements. Studies have been conducted on examining the protein and amino acid composition of certain staple foods and Nigerian diets^(11–13)^, protein and specific amino acid requirements in individuals^(2,5,9,14,15)^, as well as dietary protein intake among children^(16)^, adolescents^(17)^ and women^(18)^. However, no clear overview of both the adequacy of dietary intake of protein, in terms of quantity, in Nigerian infants, children, adolescents and (pregnant and lactating) women, and the role of the Nigerian diet and specific staple foods in achieving this adequacy exists. Hence, the present review aims at comparing the Nigerian dietary intake of protein with the protein recommendations for these groups. Furthermore, we examined the Nigerian diets regarding the most commonly eaten food groups and staple foods. We also made an effort to examine protein quality, using the most recent protein quality measure, digestible indispensable amino acid score (DIAAS)^(19)^, of some major staple foods that constitute the Nigerian diet. Protein quality was also determined for a mixture of foods since foods are often eaten together with other foods and might complement each other in terms of protein quality^(19)^.

Overall, with this article, we aim to provide a comprehensive overview within the limitations of existing data of protein quality and quantity in the diets of Nigerian infants, children, adolescents, and pregnant and lactating women.

## Methodology

The present narrative review has been conducted while applying the methodological rigour of systematic reviews as much as possible. The exact procedures for every step of the writing are described below.

### Literature search

A literature search was conducted as described below:

(1) The following database search engines were used: Scopus, PubMed, Google Scholar and the online library of the Wageningen University and Research. The following search query was used to select relevant articles about nutrition and diet in Nigerian infants, children, adolescents and women: “(nutrition* OR diet*) AND (intake) AND (Nigeria) AND (children OR women OR infants)”. Only articles published after the year 2000 were included to reflect the recent situation. This search yielded 329 articles. All abstracts were reviewed, and relevant complete articles were retrieved.
(2) Additional key words and queries such as ‘amino acids’, ‘protein’, ‘requirements’, ‘recommendations’, ‘infants’, ‘children’, ‘women’, ‘adolescents’, ‘Nigeria’, ‘diet’, ‘consumption’, ‘commonly consumed’ and ‘staple foods’, ‘protein quantity’ and ‘protein quality’ were used to categorise articles according to the different research questions, i.e. protein intake adequacy, sources of protein in Nigerian diets and protein quality.

### Protein intake adequacy

Studies assessing protein intake in terms of quantity were reviewed based on the study area and population group. The adequacy of this protein intake was calculated using the mean protein intake and the protein requirement, both estimated average requirement (EAR) and recommended daily allowance (RDA) of the participants. The EAR reported by the FAO^(19)^ and the RDA reported by WHO/FAO/UNU^(2)^ were used. The EAR is the amount of a nutrient needed to meet the needs of 50 % of the population, whereas the RDA meets the needs of almost everyone (97–98 %). These and the mean weight of the participants as reported in the studies were used to calculate the recommended daily protein intake (g/d) per population group. When no mean weight was reported in the article, a reference weight was used. The study of Walpole *et al.* conducted in 2012^(20)^ estimated an average body mass of 60·7 kg for African adults, both male and female, which was used in the present review as a reference weight when adult body weight was not reported in the study. The level of satisfaction (LOS), which is a measure of protein intake adequacy, as reported in 2014 by Akerele *et al.* ^(4)^, was calculated by dividing the mean protein intake by the calculated recommended daily protein intake for the participants (both EAR and RDA). A LOS above 100 % EAR/RDA means that the protein intake can be considered adequate. The protein intake adequacy of pregnant individuals was calculated in two ways: using an EAR/RDA based on the additional weight (kg) gained during pregnancy or using an EAR/RDA based on normal-weight individuals with an addition of extra protein requirements for pregnancy to the EAR/RDA.

### Nigerian diet and its contribution to protein intake adequacy

Major consumed foods and meals across different food groups were examined: cereals, nuts and seeds, legumes, fruits, vegetables, dairy products, meat, poultry, eggs and fish, snacks, roots and tubers, and fats and oils among different age groups. About forty articles were used in compiling an overview of Nigerian consumption patterns mostly based on 24 h recalls and food frequency questionnaires (FFQs).

### Protein quality of major staple foods

To assess the dietary protein quality of foods, we used the DIAAS. This measure is based on true ileal digestibility values of individual amino acids rather than the overall (faecal) digestibility of protein which was used in the older method of assessing protein quality: protein digestibility-corrected amino acid score (PDCAAS). Therefore, the DIAAS approach better reflects the number of amino acids absorbed^(19)^. The FAO reported that the digestibility factors should preferably be determined in human subjects, but when this is not possible it can be determined in growing pigs or rats^(21)^.

The FAO^(19)^ reported protein and amino acid intake recommendations for many age groups, but in the end gave the suggestion to use three different age ranges for amino acid scoring patterns to be used in the calculation of protein quality (DIAAS): infants (birth to 6 months), children (6 months to 3 years of age) and older children, adolescents and adults (> 3 years). This age stratification was also used in the present review (Table 1). Nine foods, mostly staples, were chosen from different food groups that were frequently eaten and had a relatively large contribution to (daily) energy or protein intake: cassava, rice, maize, wheat, yam, fish (tilapia), groundnuts, cowpeas and sorghum.

**Table 1.** Scoring patterns for calculating the digestible indispensable amino acid score in mg/g protein requirement

Age	Histidine	Isoleucine	Leucine	Lysine	SAA	AAA	Threonine	Tryptophan	Valine
Infant (0–6 months)*	21	55	96	69	33	94	44	17	55
Child (6 months–3 years)†	20	32	66	57	27	52	31	8·5	43
Older child, adolescent and adult (> 3 years)‡	16	30	61	48	23	41	25	6·6	40

SAA, sulfur amino acids (methionine and cysteine); AAA, aromatic amino acids (phenylalanine and tyrosine).

* Based on gross amino acid content in human milk from Table 4 in the FAO report^(19)^.

† Based on 0·5-year values from Table 3 in the FAO report.

‡ Based on 3- to 10-year values from Table 3 in the FAO report.

The following equation was used for calculating the DIAAS of these foods:
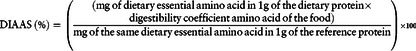
The digestibility is given as a percentage and must be divided by 100 to obtain the digestibility coefficient (for example, digestibility is 88 %, the coefficient is 88/100 = 0·88). The reference protein is based on the amino acid scoring patterns as proposed by the FAO^(19)^ (Table 1). The limiting amino acid is the amino acid with the lowest score and this is used to reflect the DIAAS of the entire food. A DIAAS ≥ 75 can be considered as a good source of protein whereas a score ≥ 100 can be considered as an excellent source of protein. The DIAAS of a mixture of foods (rice, beans and fish) was determined to examine the effects of combining multiple foods from different food groups on protein quality. Those foods were selected based on the following: availability of the DIAAS values, the fact that they reflect typical Nigerian meal compositions and aim to optimise the protein quality. The method that was used is adapted from FAO^(19)^ and is based on portion sizes of different foods, protein content, amino acid content, true ileal digestibility factors, and the three scoring patterns. Portion sizes (single servings) for rice and cowpeas were adapted from Sanusi & Olurin^(22)^ and the portion size of tilapia was set at 50 g. The amino acid content in those food portions was calculated as (amino acid content of food (mg/g protein) × true ileal digestibility factors of amino acids in the same food) × (protein content in portion per food). These were compared with references set by the FAO for different age groups to determine the DIAAS^(19)^. (The exact calculations for this mixture are explained in the footnotes of Table 11.)

## Results and discussion

### Protein intake adequacy

**Fig. 1.**
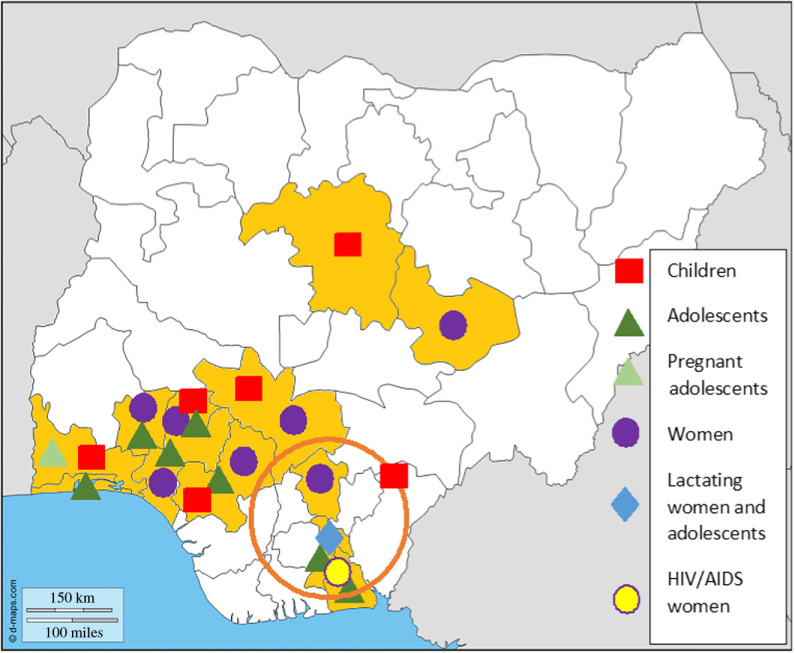
Map of Nigeria showing the individual states where protein intake was assessed, with the circle indicating a study area studied in one study rather than one state. Blank map adapted from D-Maps^(93)^.

The studies conducted in Nigeria on protein intake cover only a small part of the Nigerian population. Most data available were obtained from the richer southern parts of Nigeria (Table 2 and Fig. 1). Protein-rich foods are usually expensive and not all households have the purchasing power to acquire them^(4)^. Therefore, it can be speculated that protein deficiency is more prevalent in northern Nigeria since there are fewer financial resources available to buy protein-rich foods. Altogether twenty articles were included for examining protein intake adequacy. A total of four studies (19 %) focused on school children (aged 2–12 years), seven articles (43 %) on adolescents (aged 10–19 years), three studies (14 %) concentrated on women including pregnant and lactating women, while six articles (29 %) addressed complete households.

**Table 2.** Adequacy of protein intake in different states in Nigeria

Study	Area in Nigeria	Design/method	Study population (*n*)	Protein intake (g) (mean ± sd)	Mean weight (kg)*	EAR/RDA (g/d)†	Level of satisfaction (%)‡ EAR/RDA
^(60)^	Enugu State	Cross-sectional survey	300 Women, of which 88·5 % were aged 25–45 years	Farmers 35·8 ± 8·4 Traders 43·1 ± 5·0 Teachers 58·6 ± 13·8	53·5 52·7 61·8	35·3/44·4 34·8/43·7 40·8/51·3	101·4/80·6 123·9/98·6 143·6/114·2
^(23)^	Edo State, Kogi State	FFQ, 3-d weighed food records	Households	Females 23·0 ± 14·3 Males 25·2 ± 19·7 Children 18·9 ± 10·4	60·7 60·7 27·4	40·1/50·4 40·1/50·4 20·7/24·9	57·4/45·6 62·8/50·0 91·3/75·9
^(29)^	Ogun State	Questionnaire, weighed food intake, chemical analysis of foods	101 Pregnant adolescents 15–19 years (trimester 3)	Rural ≤17 years: 33·6 ± 2·4 Rural >17 years: 37·0 ± 3·0 Urban ≤17 years: 33·1 ± 2·2 Urban >17 years: 42·2 ± 3·9	Normal/pregnancy gain 56·4/67·4 56·4/67·4 56·4/67·4 56·4/67·4	(a) Normal (b) +24·9 pregnancy requirement (a) 64·4/47·2 (b) 78·6/56·6 (a) 64·4/47·2 (b) 78·6/56·6 (a) 64·4/47·2 (b) 78·6/56·6 (a) 64·4/47·2 (b) 78·6/56·6	(a) Normal, (b) +24·9 pregnancy requirement (a) 52·2/71·2 (b) 42·7/59·4 (a) 57·5/78·4 (b) 47·1/65·4 (a) 51·4/70·1 (b) 42·1/58·5 (a) 65·5/89·4 (b) 53·7/74·6
^(39)^	Edo State	FFQ	Normal and protein-deficient children, 3–5 years	Normal 40·3 ± 0·7 Protein-deficient 33·6 ± 1·1	16·8 16·8	12·3/15·1 12·3/15·1	327·6/266·9 273·2/222·5
^(37)^	Jos and 40 km east of Jos, Plateau State	Urban subjects: 4 d dietary recall. Rural subjects (data used from Glew *et al.* ^(61)^): 7 d dietary recall and FFQ	Urban (55 men aged 20–75 years and 77 women aged 20–70 years) and Fulani (rural) subjects (42 men and 79 women)	Males Urban 75 ± 35 Rural 85 ± 19 Females Urban 63 ± 24 Rural 62 ± 14	65·5 57·8 72·2 50·5	43·2/54·4 38·1/48·0 47·7/59·9 33·3/41·9	173·6/137·9 223·1/177·1 132·1/105·2 186·2/148·0
^(62)^	Akwa Ibom State	Not specified	418 Female adolescents aged 12–18 years	31·7 ± 7·8	51·3	36·7/44·4	86·4/71·4
^(24)^	Abia State	3 d weighed inventory	190 Adolescents, boys and girls 15–18 years	Males 54·4 ± 7·9 Females 51·5 ± 9·6	56·5 53·5	39·6/49·2 37·5/44·9	137·4/110·6 137·3/114·7
^(63)^	Uyo, Akwa Ibom State	24hR	300 Adults living with HIV and/or AIDS (160 female, 140 male)	Males 18–30 years: 34·8 ± 6·4 31–43 years: 35·9 ± 8·2 44–56 years: 36·5 ± 6·1 57–69 years: 38·8 ± 6·2 Females 18–30 years: 42·1 ± 9·3 31–43 years: 43·4 ± 12·5 44–56 years: 42·5 ± 4·8 57–69 years: –	60·7 60·7 60·7 60·7 60·7 60·7 60·7 60·7	60·1/75·6 60·1/75·6 60·1/75·6 60·1/75·6 60·1/75·6 60·1/75·6 60·1/75·6 60·1/75·6	57·9/46·0 59·7/47·5 60·7/48·3 64·6/51·3 70·0/55·7 72·2/57·4 70·7/56·2 –/–
^(25)^	Abia State	3 d weighed food intake	160 Adolescent girls 10–19 years	Secondary 93·5 University 135·4	51·3 51·3	36·7/44·4 36·7/44·4	254·8/210·6 368·9/305·0
^(26)^	Lagos State	Food intake measurements (not specified)	40 College students (adolescents) 10–19 years	10–12 years: 38·5 13–15 years: 55·0 16–19 years: 70·5 Total 56·0	54·0 59·7 59·5 58·9	39·4/48·6 43·6/52·2 41·7/50·9 41·2/52·2	97·7/79·2 126·1/105·4 169·1/138·5 135·9/107·3
^(64)^	Lagos State	Food intake measurements (not specified)	40 College students (adolescents) 11–17 years	56·0 ± 21·1	58·9	41·2/51·5	135·9/108·7
^(38)^	Orhionmwon and Ikpoba-Okha, Edo State	48-h recall method	384 Household members	Males <6 years: 20·7 6–10 years: 25·8 11–18 years: 45·8 19–59 years: 54·6 >60 years: 70·1 Females <6 years: 22·8 6–10 years: 26·2 11–18 years: 35·7 19–59 years: 42·9 >60 years: 66·7	12·7 28·1 55·8 60·7 60·7 12·0 28·5 51·3 60·7 60·7	11·3/13·7 20·5/25·3 39·9/49·4 40·1/50·4 40·1/50·4 10·7/13·0 20·8/25·7 36·7/44·4 40·1/50·4 40·1/50·4	183·2/151·1 125·9/102·0 114·8/92·7 136·2/108·3 174·8/139·1 213·1/175·4 126·0/101·9 97·3/80·4 107·0/85·1 166·3/132·3
^(13)^	Southeast Nigeria	24hR	656 Cassava-consuming children aged 2–5 years	2·5 ± 1·2 g/kg: 33	13·1	10·5/12·2	314·3/270·5
^(16)^	Ogun State	3 d weighed measurements and 24hR	116 Preschool children (24–60 months)	17·0 ± 7·2	15·2	12·2/14·2	139·3/119·7
^(65)^	Kaduna, Kaduna State	Cross-sectional and descriptive (questionnaire and 24hR)	394 School-age children 7–11 years from two schools: conventional primary school and integrated Qur’anic school	Conventional primary school 7–9 years: 23·1 ± 6·4 10–11 years: 32·7 ± 10·2 Total: 28·8 ± 10·0 Integrated Qur’anic school 7–9 years: 30·0 ± 8·3 10–11 years: 33·5 ± 9·2 Total: 32·2 ± 9·0 Total 7–9 years: 26·4 ± 8·1 10–11 years: 33·1 ± 9·7 Total: 30·5 ± 9·7	28·3 36·6 32·4 28·3 36·6 32·4 28·3 36·6 32·4	20·7/25·5 26·7/32·9 23·7/29·2 20·7/25·5 26·7/32·9 23·7/29·2 20·7/25·5 26·7/32·9 23·7/29·2	111·6/90·6 122·5/99·4 121·5/98·6 144·9/117·6 125·5/101·8 135·9/110·3 127·5/103·5 124·0/100·6 128·7/104·5
^(66)^	Ondo and Osun State	FFQ	402 University students from Obafemi Awolowo University (1) and Adekunle Ajasin University (2) aged 15–35 years	Males University 1: 84 ± 16 University 2: 83 ± 14 Females University 1: 83 ± 25 University 2: 88 ± 22	61·0 61·0 59·0 58·0	40·3/51·9 40·3/51·9 38·9/49·3 38·3/48·4	208·4/161·8 206·0/159·9 213·4/168·4 229·8/181·8
^(4)^	Ado Ekiti, in Ekiti State	Questionnaires and 24hR	321 Household members	Males < 6 years: 23·8 6–10 years: 34·1 11–18 years: 53·8 19–59 years: 65·0 Females < 6 years: 23·5 6–10 years: 30·9 11–18 years: 51·1 19–59 years: 63·7	12·7 28·1 55·8 60·7 12·0 28·5 51·3 60·7	11·3/13·7 20·5/25·3 39·9/49·4 40·1/50·1 10·7/13·0 20·8/25·7 36·7/44·4 40·1/50·4	210·6/173·7 166·3/134·8 134·8/108·9 162·1/129·7 219·6/180·8 148·6/120·2 139·2/115·1 158·9/126·4
^(67)^	Abia State	24hR and FFQ	Lactating women and adolescents 16–45 years	50·0 ± 12·2	68·4	59·1/72·8	84·6/68·7
^(36)^	Across all states of Nigeria	Data from The Nigeria Living Standard Survey 2003–2004^(68)^. Calculations based on food expenditure (collected weekly for 6 weeks) and edible portion sizes	13 142 Households	Urban 77·4 Rural 78·6	27·0 27·0	21·9/26·7 21·9/26·7	353·4/289·9 358·9/294·4
^(34)^	Iwo Local Government area of Osun State	Cross-sectional and descriptive (24hR)	250 Women, 20–59 years	56 ± 84	56·8	37·5/47·1	149·3/118·9
^(69)^	Across all states of Nigeria	Data from General Household Survey 2010–2011^(70)^. Food consumption past 7 d. Calculations based on household food expenditure and food prices	1557 Rural households	Post-harvest season 147·0 Post-planting season 117·4	27·0 27·0	21·9/26·7 21·9/26·7	671·2/550·6 536·1/439·7

EAR§ (RDA║) protein	
Age (years)	g/kg per d
0·5	1·12 (1·31)
1	0·86 (1·14)
2	0·86 (0·97)
3–10	0·73 (0·90)
11–14	0·73 (0·90 male/0·89 female)
15–18	0·70 (0·87 male/0·84 female)
>18	0·66 (0·83)
Pregnancy║ Trimester 1 Trimester 2 Trimester 3 HIV║ Lactating║ ≤6 months >6 months	+0·5 (0·7) g/d +7·7 (9·6) g/d +24·9 (31·2) g/d 50 % allowance 15·1 (18·9) 10·0 (12·5)

Reference weights (when actual weight unknown)║		
Age (years)	Male (kg)	Female (kg)
0·5	7·8	7·2
1	10·2	9·5
1·5	11·5	10·8
2	12·3	11·8
3	14·6	14·1
4–6	19·7	18·6
7–10	28·1	28·5
11–14	45·0	46·1
15–18	66·5	56·4
>18¶	60·7	60·7
Pregnancy Trimester 1 Trimester 2 Trimester 3		+0·8 +4·8 +11·0

EAR, estimated average requirement; FFQ, food frequency questionnaire; RDA, recommended daily allowance; 24hR, 24 h recall.

* Mean weight expressed in kg, weight used as reported in an article if available, if not a reference weight was used. Average reference weights were calculated when a study covered multiple age groups. When only household data were available, a mean weight of 27·0 kg was calculated from all the reference weights for men and (non-pregnant) women for all ages.

† EAR = sum of protein needed for maintenance and growth^(19)^ = ‘the intake that meets the estimated nutrient needs of half the individuals in a group’. RDA (safe intake level) = average protein requirement plus twice the standard deviation, meeting the needs of 97–98 % of the population^(2)^.

‡ Level of satisfaction (%) calculated as protein in g/d consumed by study population divided by the calculated EAR/RDA in g/d × 100.

§ Adapted from FAO^(19)^.

║ Adapted from WHO/FAO/UNU^(2)^.

¶ Adapted from Walpole *et al.* ^(20)^.

It is important to realise the limitations of the present review due to the quality of the reported data. The protein intake values as reported in our review were mean protein intake values. These values are valid to determine average protein intake adequacy; however, they do not take a distribution of intake into account. Furthermore, studies conducted on Nigerian pregnant and lactating individuals were scarce. Due to lack of data (we found only one study; Table 2) on the protein intake among diseased (for example, HIV/AIDS, respiratory diseases, etc.) target populations, we did not analyse this further. Some studies did report a percentage of stunted/wasted children, but most gave no separate protein intake value for this group. Other studies did not provide information on whether the study population included pregnant, lactating or diseased individuals. Also, many studies did not report the actual weights of the participants, making it difficult to calculate a precise EAR/RDA based on their actual body weight.

Based on the reviewed literature, protein intake of the Nigerian population of non-pregnant and non-lactating women and children seems to be mostly adequate (LOS > 100 %). However, notable exceptions were also reported in the study on overall households’ intakes in Edo and Kogi, where protein intake was reported to be inadequate for all family members, with females’ intakes being the lowest^(23)^. Based on our calculations, protein intake of female adolescents seems to be mostly inadequate (LOS < 100 %). The exceptions of satisfactory intake were reported by Ogechi *et al.* ^(24)^ and Anyika *et al.* ^(25)^, and for adolescents above 13 years by Akinyemi & Ibraheem^(26)^. Pregnant and lactating women were another group of concern with inadequate protein intake. This could be due to cultural beliefs that exist in certain parts of Nigeria that extra protein cannot be consumed during pregnancy^(27)^ and myths about some forbidden protein-rich foods during pregnancy (for example, eggs, beans, snails and grasscutter meat)^(28)^. Especially, the protein intake of pregnant adolescents was low (LOS_EAR_ < 70 %^(29)^); these females were not even able to meet the requirements for healthy non-pregnant and non-lactating women (LOS_EAR_ < 84·0 %; and LOS_RDA_ < 69·8 %). The adult lactating women could only just meet the EAR (LOS_EAR_ = 104 %), but not the RDA (LOS_RDA_ = 86·1 %) for healthy non-pregnant and non-lactating women. This is of concern because inadequate protein intake during pregnancy has both short- and long-term consequences for both the infant and mother. Epidemiological studies in human subjects have shown that protein deficiency during pregnancy gives rise to low birth weight, intra-uterine growth restriction and that these offspring are at greater risk for development of the metabolic syndrome in adult life^(30)^. Nutritional intervention during pregnancy is necessary to ensure that mothers consume appropriate amounts of dietary protein such that there are no negative effects on fetal growth and development^(30)^. Adequate nutrition is also important during the lactation period, as the nutritional content of breast milk was shown to be dependent on maternal diet, for example, fatty acid content, water-soluble vitamins, etc. There are also some reports indicating that the protein quality of the mother’s diet is reflected in the amino acid composition of the breast milk^(31,32)^. Especially lysine^(31,32)^, methionine^(32)^ and tryptophan^(31)^ were substantially reduced in women with poor protein intake from the diet (for example, by consumption of mostly cereal grains and legumes).

Interestingly, sufficient protein intakes (LOS > 200) were also reported among children classified as protein deficient based on their anthropomorphic measures^(13)^. When studies provided their own level of adequacy and reported percentages of individuals with inadequate intakes, a more detailed picture emerged. For example, in the only study with children (2–5 years old) in which such distinction was made, 13 % of the participants were reported to have inadequate protein intake^(13)^. Within studies focused on women, inadequate intakes were reported for 18 % of women of childbearing age (15–49 years) in south-east Nigeria^(33)^, 14·4 % of women aged 20–59 years in Osun State^(34)^, and 22·2 % of adolescent females in Osun^(17)^.

### Nigerian diet and its contribution to protein intake adequacy

Previous literature concluded that dietary diversity was low in six states in Nigeria and should be increased^(35)^. The search into the Nigerian consumption pattern (Table 3; fruits, vegetables, oils and fats, and snacks not shown) shows that all examined food groups in the Nigerian diet were consumed frequently by at least 25 % of the population, with ‘fats and oils’, ‘cereals’ and ‘snacks’ being consumed the most (> 65·0 %). Snack consumption was most often reported in studies assessing adolescents and could be an explanation for the low protein intake of adolescent girls since snacks are often fat- and carbohydrate-rich, but protein-poor. Wheat and rice were the most frequent consumed cereals. Cassava and yam were the most eaten roots and tubers; beans and groundnuts the most frequently consumed legumes. Beef, chicken and fish were frequently eaten animal foods, while dairy products were less frequently consumed.

**Table 3.** Commonly consumed foods and meals from different food groups and their contribution to meeting the protein requirements

Commonly eaten foods/dishes	Population group	States in Nigeria	% of consumption
Roots and tubers			
Whole group	Households, adolescents, women, infants, children, lactating and pregnant women, and adolescents	Nationwide, Osun, Ondo, Borno, Kaduna, Taraba, Kwara, Akwa Ibom, Abia	78·3^(70)^, 45·5^(66)^, 17·6^(3)^, 74·4^(34)^, 59·8^(35)^, 34·5^(67)^, 36·2^(78)^ Average 49·5
Yam	Adolescents, households, infants, children, women, pregnant adolescents	Kwara, nationwide, Osun, Federal Capital Territory Abuja, Enugu, Ogun	11·3^(74)^, 50·0^(46)^, 9·0^(46)^, 30·2^(44)^, 7·6^(53)^, 34·8^(79)^, 9·1^(79)^, 25·0^(79)^, 5·5^(79)^ Average 20·3
Cocoyam	Households	Nationwide	15·0^(46)^
Cassava	Households, adolescents, children	Nationwide, Kwara, Enugu, South-East	20·0^(46)^, 50·0^(46)^, 11·3^(74)^ Average 27·1
Potatoes	Adolescents, households, infants, children	Kwara, nationwide, Osun, Enugu	11·3^(74)^, 6·0^(46)^, 14·0^(46)^, 0·9^(44)^, 3·0^(79)^ Average 7·0
Meals/dishes			
*Amala* (made with yam flour)	Infants, women, children	Osun, Federal Capital Territory Abuja	Average 22·1^(44,53)^
Pigeon pea pottage + cassava	Children, adolescents	Enugu	5·05^(79)^
*Fufu*	Infants, women, children, adolescents	Osun, Federal Capital Territory Abuja, Enugu	Average 15·5^(44,53,79)^
*Eba* (made from *garri*)	Infants, women, children	Osun, Federal Capital Territory Abuja	Average 21·0^(44,53)^
*Akpu* (cassava-based porridge)	Women, children, adolescents	Federal Capital Territory Abuja, Enugu	Average 10·0^(53,79)^
*Garri* (cassava flower)	Children, adolescents	Enugu	8·0^(79)^
Legumes, nuts and seeds			
Legumes	Adolescents, infants, children, women, pregnant and lactating women, and adolescents	Osun, nationwide, Plateau, Borno, Kaduna, Taraba, Kwara, Akwa Ibom, Ondo, Abia, Ondo Abia, Cross River	52·2^(17)^, 23·0^(3)^, 70·4^(34)^, 77·0^(37)^, 63·5^(35)^, 47·5^(77)^, 21·40^(67)^, 35·7^(78)^, 18·6^(81)^ Average 45·5
Nuts and seeds	Households, women, infants, children, pregnant and lactating women, and adolescents	Nationwide, Osun, Borne, Kaduna, Taraba, Kawar, Aqua Ibom, Ondo, Abiu, Cross River	77·2^(70)^, 26·8^(34)^, 23·0^(3)^, 63·5^(35)^, 35·7^(78)^, 18·6^(81)^ Average 40·8
Beans	Households, infants, children, adolescents	Nationwide, Osun, Ondo, Oyo, Enugu	68·0^(46)^, 24·4^(44)^, 29·5^(80)^, 9·1^(79)^ Average 32·8
Peas	Households, children, adolescents	Nationwide, Enugu	13·0^(46)^, 5·5^(79)^ Average 9·3
Palm kernel	Children, adolescents	Enugu	5·5^(79)^
Groundnuts	Households, children, adolescents, women, infants	Nationwide, Enugu, Kwara, Oyo, Ondo	33·0^(46)^, 8·1^(79)^, 11·3^(74)^, 35·3^(80)^ Average 21·9
Meals/dishes			
*Akara* (made with cowpeas)	Infants, adolescents, children	Osun, Enugu	23·1^(44)^, 3·0^(79)^ Average 13·1
Bean porridge	Children, adolescents	Enugu	6·5^(79)^
*Ayaraya oka* (cowpeas and maize)	Children, adolescents	Enugu	11·0^(79)^
*Moin-moin* (made with peas)	Infants, children, adolescents	Osun, Enugu	28·4^(44)^, 2·0^(79)^ Average 15·2
*Okpa* (steamed *bambara* groundnut paste pudding)	Children, adolescents	Enugu	25·2^(79)^
Dairy products			
Whole group	Children, households, adolescents, infants, women, pregnant and lactating adolescents, and women	South-East, nationwide, Osun, Kwara, Borno, Kaduna, Taraba, Akwa Ibom, Cross River	41·0^(.13)^, 90·8^(70)^, 38·0^(70)^, 38·7^(17)^, 14·6^(3)^, 10·8^(34)^, 27·0^(35)^, 17·4^(78)^, 67·6^(81)^ Average 38·4
Milk (all sorts)	Women, adolescents, infants, children, pregnant and lactating women and adolescents, households	Osun, Ondo, Nationwide, Kwara, Plateau, Oyo	20·5^(66)^, 13·5^(3)^, 2·7^(74)^, 87·0^(37)^, 26·9^(78)^, 47·6^(44)^, 47·6^(80)^, 50·5^(80)^, 11·0^(46)^, 22·0^(46)^, 21·8^(44)^ Average 31·9
Cheese	Adolescents, infants, children	Kwara, Osun	16·1^(74)^, 4·0^(44)^ Average 10·1
Ice cream/(frozen) yoghurt	Adolescents, children	Kwara, Oyo, Enugu	7·2^(74)^, 48·5^(80)^, 2·0^(79)^, 24·1^(80)^ Average 20·5
Meat, poultry, eggs and fish			
Meat and poultry products	Women, adolescents, infants, children, lactating women and adolescents, households	Osun, Ondo, nationwide, Kwara, Plateau, Borno, Kaduna, Taraba, Osun, Akwa Ibom, Abia, Cross River, South-East	40·0^(66)^, 48·8^(17)^, 31·4^(3)^, 9·9^(74)^, 33·0^(78)^, 41·2^(34)^, 100·0^(61)^, 92·0^(37)^, 40·0^(44)^, 33·4^(35)^, 47·5^(77)^, 34·3^(67)^, 44·1^(81)^, 41·0^(13,73)^, 90·8^(70)^ Average 48·5
Beef	Households, adolescents	Niger, Lagos, nationwide, Oyo	47·0^(72)^, 55·0^(46)^, 29·9^(80)^, 31·0^(80)^ Average 40·7
Pork	Households, adolescents	Nationwide, Oyo, Kwara	1·0^(46)^, 17·3^(80)^, 6·4^(74)^ Average 8·2
Goat/mutton	Households	Niger, Lagos, nationwide	20·0^(72)^, 15·0^(46)^ Average 17·5
Chicken	Households, adolescents, women	Niger, Lagos, nationwide, Oyo, Osun	14·0^(72)^, 5·0^(46)^, 33·7^(80)^, 41·2^(80)^, 31·7^(80)^, 27·2^(34)^ Average 25·5
Organ meat	Adolescents, infants, children, pregnant and lactating women, and adolescents	Ondo, nationwide	4·2^(77)^, 12·2^(78)^ Average 8·2
Grasscutter	Households	Niger, Lagos	9·0^(72)^
Eggs	Infants, children, adolescents, women, households, pregnant and lactating women and adolescents	Nationwide, Kwara, Osun, Plateau, Borno, Kaduna, Taraba, Akwa Ibom, Cross River, Oyo	15·8^(3)^, 5·0^(74)^, 27·2^(34)^, 87·0^(37)^, 10·0^(46)^, 39·1^(44)^, 4·6^(35)^, 16·9^(78)^, 9·6^(81)^, 49·2^(80)^ Average 26·4
Fish and shellfish	Women, adolescents, infants, children, pregnant and lactating women and adolescents, households	Osun, Plateau, Borno, Kaduna, Taraba, Kwara, Osun, Akwa Ibom, Ondo, nationwide, Oyo, South-East	52·4^(34)^, 60·0^(61)^, 86·0^(37)^, 48·9^(44)^, 57·1^(35)^, 70·0^(77)^, 42·8^(78)^, 31·1^(80)^, 49·2^(80)^, 35·1^(80)^, 48·8^(17)^, 31·4^(3)^, 9·9^(74)^, 41·0^(13,73)^, 90·8^(70)^, 5·0^(46)^, 65·0^(46)^ Average 49·1
Tilapia	Households	Lagos, Niger	19·0–31·0^(72)^
Crustaceans/molluscs	Households	Nationwide	23·0^(46)^
Meals/dishes			
Meat pies	Adolescents	Oyo	37·8^(80)^
Burgers/hotdogs	Adolescents	Kwara	14·8^(74)^
Cereals/grains			
Whole group	Households, women, adolescents, infants, children, pregnant and lactating women, and adolescents	Nationwide, Osun, Ondo, Borno, Kaduna, Taraba, Kwara, Akwa Ibom, Abia, Cross River	96·7^(70)^, 63·5^(66)^, 76·5^(17)^, 61·3^(3)^, 98·0^(34)^, 100·0^(37)^, 92·1^(35)^, 75·0^(77)^, 67·6^(67)^, 72·8^(78)^, 91·0^(81)^ Average 81·3
Rice	Households, infants, children, women, adolescents, pregnant adolescents	Nationwide, Osun, Federal Capital Territory Abuja, Kwara, Enugu, Oyo, Ogun	76·0^(46)^, 48·9^(44)^, 31·5^(53)^, 15·0^(74)^, 87·8^(79)^, 28·9^(80)^, 71·3^(80)^ Average 51·3
Wheat	Preschool children, households	Southeast Nigeria, nationwide	58·0^(46)^
Maize	Adolescents, households, women, children, infants	Kwara, nationwide, Federal Capital Territory Abuja, Enugu, Oyo, Nasarawa	4·7^(74)^, 46·0^(46)^, 4·4^(53)^, 2·0^(79)^, 36·9^(80)^ Average 18·8
Pasta (for example, spaghetti, noodles)	Adolescents, households, infants, children	Kwara, nationwide, Osun, Enugu, Oyo	6·0^(74)^, 12·0^(46)^, 15·6^(44)^, 1·5^(79)^, 3·0^(79)^, 51·8^(80)^ Average 14·9
Bread	Households, infants, women, children and adolescents	Nationwide, Federal Capital Territory Abuja, Enugu, Oyo	40·9^(44)^, 26·8^(53)^, 2·5^(79)^, 7·0^(79)^, 58·1^(80)^ Average 27·1
Breakfast cereal (for example, cornflakes, Golden Morn)	Infants, children, pregnant and lactating adolescents and women, adolescents	Osun, nationwide, Oyo	8·0^(44)^, 6·6^(44)^, 6·6^(44)^, 5·5^(78)^, 31·4^(80)^, 27·8^(80)^ Average 14·3
Sorghum	Households	Nationwide	44·0^(46)^
Millet	Households	Nationwide	34·0^(46)^
Oat bran (meal)	Adolescents	Kwara, Oyo	5·8^(74)^, 11·2^(80)^ Average 8·5
Meals/dishes			
*Ayaraya oka* (cowpeas and maize)	Children, adolescents	Enugu	11·0^(79)^
*Jollof* rice	Adolescents	Oyo	42·3^(80)^
Semovita (wheat-based porridge)	Women	Federal Capital Territory Abuja	11·8^(53)^
Pancakes	Adolescents	Oyo	19·5^(80)^
Pap	Infants, children, pregnant and lactating women and adolescents, adolescents, women	Osun, nationwide, Oyo, Nasarawa, Borno, Kaduna, Taraba, Kwara, Akwa Ibom, Kebbi, Niger	34·7^(44)^, 15·6^(44)^, 58·0^(44)^, 53·0^(78)^, 24·5^(80)^, 29·0^(80)^ Average 35·8

The percentage of the Nigerian population that frequently consumed roots and tubers is 49·5 %. Cereals and grains were on average consumed daily by 81·3 % of the population. Several studies showed a contribution of cereals to the protein intake of approximately 20–40 %^(36–38)^. Legumes were eaten by 45·5 % of the population on average.

Meat, poultry and fish intakes varied widely depending on the study and geographical location, but on average the consumption percentage was 48·5 % for meat and poultry and 49·1 % for fish and shellfish. According to a study conducted in the south-east of Nigeria, animal-source products contributed only 3 % to the total daily energy intake^(13)^. Dairy products were found to account for barely 0·9 % of protein intake^(36)^. In general, dairy products were only consumed by 38·4 % of the population, with milk being consumed by only 31·9 % (Table 3).

### Protein quality of staple foods

Even though most Nigerian population groups seem to have adequate protein intakes in terms of quantity, it does not guarantee high-enough protein quality. The drastic effects of a poor protein quality cassava-based diet were demonstrated in a study among children displaying signs of protein malnutrition despite their overall protein intakes being very high with LOS > 200^(39)^. However, since almost complete protein intake was based on cassava, which has very low protein quality, with leucine being the first limiting amino acid (DIAAS 11–18), it did not cover the children’s nutritional needs, resulting in malnutrition. Overall, the nine major staple foods eaten by Nigerians appear not to deliver protein of good quality (Tables 4–9). For the foods from the cereal group (rice, wheat, maize and sorghum), lysine was the first limiting amino acid for all age groups, resulting in DIAAS being inadequate (< 75). Wheat and rice were shown to be of good protein quality for children and adults when looking at the second limiting amino acids (DIAAS > 75), but not for infants. The digestibility factor we used for calculating DIAAS of yam was the overall crude protein digestibility for cassava since no factor for yam could be found in the literature. Protein quality of yam depended on the way of its processing. Processed yam compared with the two unprocessed species had lower protein quality in terms of limiting amino acids (DIAAS < 75). The unprocessed (and thus uneatable) yam was shown to be of (marginally) better protein quality for the first SAA and second (lysine) limiting amino acids (DIAAS > 75 for population > 3 years of age). For both groundnuts and cowpeas, the first limiting amino acid was also lysine (DIAAS 27–38), the second SAA (groundnut DIAAS 40–58; cowpea DIAAS 33–49). The DIAAS of rice, maize, wheat and sorghum were, respectively, 42–69, 37–53, 30–43 and 18–26. All sources of cereals/grains did not have good-quality proteins based on their amino acid score and digestibility, and the most deficient amino acid appears to be lysine, followed by SAA. The only source of good-quality protein, which is commonly consumed, was fish. Tilapia, which is also frequently eaten in Nigeria, was the only fish for which information was available to calculate DIAAS: DIAAS > 94 for children 6 months–3 years of age, and excellent DIAAS > 100 for individuals above 3 years.

**Table 4.** Digestible indispensable amino acid score (DIAAS) of cassava and rice*

Essential amino acids	Cassava					Rice				
	AA (mg/g protein)	True ileal digestibility†	Reference ratio infants‡	Reference ratio children	Reference ratio other	AA (mg/g protein)	True ileal digestibility	Reference ratio infants	Reference ratio children	Reference ratio other
Histidine	38·5		1·58	1·66	2·07	23	0·99	1·08	1·14	1·42
Isoleucine	9·3		0·15	0·25	0·27	35	0·75	0·48	0·82	0·88
Leucine	12·8		0·11	0·17	0·18	77	0·81	0·65	0·95	1·02
Lysine	17·6		0·22	0·27	0·32	36	0·92	0·48	0·58	0·69
SAA	25·7		0·67	0·82	0·96	53	0·92	1·47	1·80	2·11
AAA	19·6		0·18	0·32	0·41	91	0·83	0·80	1·44	1·83
Threonine	15·9		0·31	0·44	0·55	34	0·82	0·63	0·90	1·12
Tryptophan						8	0·89	0·42	0·84	1·08
Valine	23·7		0·37	0·47	0·51	57	0·88	0·91	1·17	1·25
Crude protein		0·86					0·9			
Source	^(83)^	^(84)^ (Pig)				^(71)^	^(85)^ (Human)			
DIAAS (%) first limiting AA§			11 (Leu)║	17 (Leu)	18 (Leu)			42 (Tryp)	58 (Lys)	69 (Lys)
DIAAS (%) second limiting AA			15 (Ileu)	25 (Ileu)	27 (Ileu)			48 (Ileu + Lys)	82 (Ileu)	88 (Ileu)
DIAAS (%) third limiting AA			18 (AAA)	27 (Lys)	32 (Lys)			63 (Thr)	84 (Tryp)	102 (Leu)

AA, amino acid; SAA, sulfur amino acids (methionine and cysteine); AAA, aromatic amino acids (phenylalanine and tyrosine).

* Adapted from FAO^(19)^.

† True ileal digestibility = ‘the disappearance of a nutrient between the mouth and the end of the small intestine (terminal ileum)’^(19)^.

‡ Reference ratio calculated as (amino acid content × digestibility)/scoring pattern.

§ Limiting AA calculated as the amino acid(s) with the lowest (red) reference ratio score × 100 %. Also done for the second (orange) and third (yellow) amino acids.

║ Essential amino acids: histidine (His), isoleucine (Ileu), leucine (Leu), lysine (Lys), methionine (Met), cysteine (Cys), phenylalanine (Phe), tyrosine (Tyr), threonine (Thr), tryptophan (Tryp) and valine (Val).

**Table 5.** Digestible indispensable amino acid score (DIAAS) of maize and wheat*

Essential amino acids	Maize					Wheat				
	AA (mg/g protein)	True ileal digestibility†	Reference ratio infants‡	Reference ratio children	Reference ratio other	AA (mg/g protein)	True ileal digestibility	Reference ratio infants	Reference ratio children	Reference ratio other
Histidine	30·7	0·81	1·18	1·24	1·55	22	0·88	0·92	0·97	1·21
Isoleucine	37·6	0·84	0·57	0·99	1·05	29	0·88	0·46	0·80	0·85
Leucine	125·2	0·88	1·15	1·67	1·81	65	0·88	0·60	0·87	0·94
Lysine	34	0·75	0·37	0·45	0·53	26	0·80	0·30	0·36	0·43
SAA	17·3	0·90	0·47	0·58	0·68	41	0·88	1·09	1·34	1·57
AAA	51·6	0·83	0·46	0·82	1·04	70	0·88	0·66	1·18	1·50
Threonine	38·4	0·78	0·68	0·97	1·20	28	0·83	0·53	0·75	0·93
Tryptophan	5·9	0·80				12	0·88	0·62	1·24	1·60
Valine	50·5	0·85	0·78	1·00	1·07	43	0·85	0·66	0·85	0·91
Crude protein							0·87			
Source	^(86)^	^(87)^ (Pig)				^(71)^	^(85)^ (Human)			
DIAAS (%) first limiting AA§			37 (Lys)║	45 (Lys)	53 (Lys)			30 (Lys)	36 (Lys)	43 (Lys)
DIAAS (%) second limiting AA			46 (AAA)	58 (SAA)	68 (SAA)			46 (Ileu)	75 (Thr)	85 (Ileu)
DIAAS (%) third limiting AA			47 (SAA)	82 (AAA)	104 (AAA)			53 (Thr)	80 (Ileu)	91 (Val)

AA, amino acid; SAA, sulfur amino acids (methionine and cysteine); AAA, aromatic amino acids (phenylalanine and tyrosine).

* Adapted from FAO^(19)^.

† True ileal digestibility = ‘the disappearance of a nutrient between the mouth and the end of the small intestine (terminal ileum)’^(19)^.

‡ Reference ratio calculated as (amino acid content × digestibility)/scoring pattern.

§ Limiting amino acid calculated as the amino acid(s) with the lowest (red) reference ratio score × 100 %. Also done for the second (orange) and third (yellow) amino acids.

║ Essential amino acids: histidine (His), isoleucine (Ileu), leucine (Leu), lysine (Lys), methionine (Met), cysteine (Cys), phenylalanine (Phe), tyrosine (Tyr), threonine (Thr), tryptophan (Tryp) and valine (Val).

**Table 6.** Digestible indispensable amino acid score (DIAAS) of yam*

	AA (mg/g protein) *Dioscorea alata* (1)	AA (mg/g protein) *D. rotundata* (2)	AA (mg/g protein) processed *D. rotundata* (3)	True ileal digestibility†	Reference ratio infants‡			Reference ratio children			Reference ratio other		
Essential amino acids					1	2	3	1	2	3	1	2	3
Histidine	23·9	26	24·5		0·98	1·06	1·00	1·03	1·12	1·05	1·28	1·40	1·32
Isoleucine	41	37·7	31·6		0·64	0·59	0·49	1·10	1·01	0·85	1·18	1·08	0·91
Leucine	89·6	83·6	40·4		0·80	0·75	0·36	1·17	1·09	0·53	1·26	1·18	0·57
Lysine	46·3	47·3	43·3		0·58	0·59	0·54	0·70	0·71	0·65	0·83	0·85	0·78
SAA	19·7	20·3	42·3		0·51	0·53	1·10	0·63	0·65	1·35	0·74	0·76	1·58
AAA	119·1	116·8	49·3		1·09	1·07	0·45	1·97	1·93	0·82	2·50	2·45	1·03
Threonine	51·5	46·9	23·1		1·01	0·92	0·45	1·43	1·30	0·64	1·77	1·61	0·79
Tryptophan	10	9·0			0·51	0·46		1·01	0·91		1·30	1·17	
Valine	46·9	41·8	27·8		0·73	0·65	0·43	0·94	0·84	0·56	1·01	0·90	0·60
Crude protein				0·86									
Source	^(88)^	^(88)^	^(89)^	Same as cassava used (pig)									
DIAAS (%) first limiting AA§					51 (SAA + Tryp)║	46 (Tryp)	36 (Leu)	63 (SAA)	65 (SAA)	53 (Leu)	74 (SAA)	76 (SAA)	57 (Leu)
DIAAS (%) second limiting AA					58 (Lys)	53 (SAA)	43 (Val)	70 (Lys)	71 (Lys)	56 (Val)	83 (Lys)	85 (Lys)	60 (Val)
DIAAS (%) third limiting AA					64 (Ileu)	59 (Lys+Ileu)	45 (AAA + Thr)	94 (Val)	84 (Val)	64 (Thr)	101 (Val)	90 (Val)	78 (Lys)

AA, amino acid; SAA, sulfur amino acids (methionine and cysteine); AAA, aromatic amino acids (phenylalanine and tyrosine).

* Adapted from FAO^(19)^.

† True ileal digestibility = ‘the disappearance of a nutrient between the mouth and the end of the small intestine (terminal ileum)’^(19)^.

‡ Reference ratio calculated as (amino acid content × digestibility)/scoring pattern.

§ Limiting amino acid calculated as the amino acid(s) with the lowest (red) reference ratio score × 100 %. Also done for the second (orange) and third (yellow) amino acids.

║ Essential amino acids: histidine (His), isoleucine (Ileu), leucine (Leu), lysine (Lys), methionine (Met), cysteine (Cys), phenylalanine (Phe), tyrosine (Tyr), threonine (Thr), tryptophan (Tryp) and valine (Val).

**Table 7.** Digestible indispensable amino acid score (DIAAS) of fish (tilapia) and groundnuts*

Essential amino acids	Fish (tilapia)					Groundnuts				
	AA (mg/g protein)	True ileal digestibility†	Reference ratio infants‡	Reference ratio children	Reference ratio other	AA (mg/g protein)	True ileal digestibility	Reference ratio infants	Reference ratio children	Reference ratio other
Histidine	23	0·85	0·93	0·98	1·22	23	0·90 (not detoxified)	0·99	1·04	1·29
Isoleucine	37	0·93	0·63	1·08	1·15	38	0·83	0·57	0·99	1·05
Leucine	72	0·91	0·68	0·99	1·07	67	0·86	0·60	0·87	0·94
Lysine	77	0·93	1·04	1·26	1·49	30	0·61	0·27	0·32	0·38
SAA	40	0·91 (crude protein)	1·10	1·35	1·58	18	0·74	0·40	0·49	0·58
AAA	69	0·83	0·61	1·10	1·40	75	0·90	0·72	1·30	1·65
Threonine	43	0·95	0·93	1·32	1·63	26	0·71	0·42	0·60	0·74
Tryptophan	14	0·91 (crude protein)	0·75	1·50	1·93		0·72			
Valine	45	0·9	0·74	0·94	1·01		0·81			
Crude protein		0·91					0·77			
Source	^(71)^	^(85)^ (Chinese fish) (human)				^(76)^	^(90)^ (Detoxified groundnut meal) (pig)			
DIAAS (%) first limiting AA§			61 (AAA)║	94 (Val)	101 (Val)			27 (Lys)	32 (Lys)	38 (Lys)
DIAAS (%) second limiting AA			63 (Ileu)	98 (His)	107 (Leu)			40 (SAA)	49 (SAA)	58 (SAA)
DIAAS (%) third limiting AA			68 (Leu)	99 (Leu)	115 (Ileu)			42 (Thr)	60 (Thr)	74 (Thr)

AA, amino acid; SAA, sulfur amino acids (methionine and cysteine); AAA, aromatic amino acids (phenylalanine and tyrosine).

* Adapted from FAO^(19)^.

† True ileal digestibility = ‘the disappearance of a nutrient between the mouth and the end of the small intestine (terminal ileum)’^(19)^.

‡ Reference ratio calculated as (amino acid content × digestibility)/scoring pattern.

§ Limiting amino acid calculated as the amino acid(s) with the lowest (red) reference ratio score × 100 %. Also done for the second (orange) and third (yellow) amino acids.

║ Essential amino acids: histidine (His), isoleucine (Ileu), leucine (Leu), lysine (Lys), methionine (Met), cysteine (Cys), phenylalanine (Phe), tyrosine (Tyr), threonine (Thr), tryptophan (Tryp) and valine (Val).

**Table 8.** Digestible indispensable amino acid score (DIAAS) of cowpeas*

	AA (mg/g protein) cream coat moderate cowpea (1)	AA (mg/g protein) white coat small cowpea (2)	True ileal digestibility†	Reference ratio infants‡		Reference ratio children		Reference ratio other	
Essential amino acids				1	2	1	2	1	2
Histidine	20·0	20·6	0·81	0·77	0·79	0·81	0·83	1·01	1·04
Isoleucine	30·8	30·2	0·77	0·43	0·42	0·74	0·73	0·79	0·78
Leucine	59·3	57·2	0·77	0·48	0·46	0·69	0·67	0·75	0·72
Lysine	28·0	29·1	0·81	0·33	0·34	0·40	0·41	0·47	0·49
SAA	21·1	20·6	0·74	0·47	0·46	0·58	0·56	0·68	0·66
AAA	63·3	69·0	0·77	0·52	0·57	0·94	1·02	1·19	1·30
Threonine	22·9	22·9	0·74	0·39	0·39	0·55	0·55	0·68	0·68
Tryptophan			0·69						
Valine	31·2	31·2	0·74	0·42	0·42	0·54	0·54	0·58	0·58
Crude protein			0·78						
Source	^(91)^	^(91)^	^(85)^ (Human)						
DIAAS (%) first limiting AA§				33 (Lys)║	34 (Lys)	40 (Lys)	41 (Lys)	47 (Lys)	49 (Lys)
DIAAS (%) second limiting AA				39 (Thr)	39 (Thr)	54 (Val)	54 (Val)	58 (Val)	58 (Val)
DIAAS (%) third limiting AA				42 (Val)	42 (Val+Ileu)	55 (Thr)	55 (Thr)	68 (SAA + Thr)	66 (SAA)

AA, amino acid; SAA, sulfur amino acids (methionine and cysteine); AAA, aromatic amino acids (phenylalanine and tyrosine).

* Adapted from FAO^(19)^.

† True ileal digestibility = ‘the disappearance of a nutrient between the mouth and the end of the small intestine (terminal ileum)’^(19)^.

‡ Reference ratio calculated as (amino acid content × digestibility)/scoring pattern.

§ Limiting amino acid calculated as the amino acid(s) with the lowest (red) reference ratio score × 100 %. Also done for the second (orange) and third (yellow) amino acids.

║ Essential amino acids: histidine (His), isoleucine (Ileu), leucine (Leu), lysine (Lys), methionine (Met), cysteine (Cys), phenylalanine (Phe), tyrosine (Tyr), threonine (Thr), tryptophan (Tryp) and valine (Val).

**Table 9.** Digestible indispensable amino acid score (DIAAS) of sorghum*

Essential amino acids	AA (mg/g protein)	True ileal digestibility†	Reference ratio infants‡	Reference ratio children	Reference ratio other
Histidine	17	0·81	0·66	0·69	0·86
Isoleucine	34	0·87	0·54	0·92	0·99
Leucine	124	0·88	1·14	1·65	1·79
Lysine	16	0·79	0·18	0·22	0·26
SAA	15	0·83	0·38	0·46	0·54
AAA	80	0·87	0·74	1·34	1·70
Threonine	31	0·84	0·59	0·84	1·04
Tryptophan	8	0·87	0·41	0·82	1·05
Valine	43	0·86	0·67	0·86	0·92
Crude protein		0·83			
Source	^(92)^	^(85)^ (Human)			
DIAAS (%) first limiting AA§			18 (Lys)║	22 (Lys)	26 (Lys)
DIAAS (%) second limiting AA			38 (SAA)	46 (SAA)	54 (SAA)
DIAAS (%) third limiting AA			41 (Tryp)	69 (His)	86 (His)

AA, amino acid; SAA, sulfur amino acids (methionine and cysteine); AAA, aromatic amino acids (phenylalanine and tyrosine).

* Adapted from FAO^(19)^.

† True ileal digestibility = ‘the disappearance of a nutrient between the mouth and the end of the small intestine (terminal ileum)’^(19)^.

‡ Reference ratio calculated as (amino acid content × digestibility)/scoring pattern.

§ Limiting amino acid calculated as the amino acid(s) with the lowest (red) reference ratio score × 100 %. Also done for the second (orange) and third (yellow) amino acids.

║ Essential amino acids: histidine (His), isoleucine (Ileu), leucine (Leu), lysine (Lys), methionine (Met), cysteine (Cys), phenylalanine (Phe), tyrosine (Tyr), threonine (Thr), tryptophan (Tryp) and valine (Val).

Overall, all DIAAS for the scoring pattern for infants were inadequate. However, infants are supposed to be exclusively breastfed, and the protein of human milk meets all the amino acid requirements. Therefore the FAO^(40)^ based the scoring pattern for infants on breast milk and consequently no foods from either plant- or animal-based foods can satisfy infant amino acid requirements like human breast milk. However, given the worrying possibility that maternal amino acid deficiencies may be reflected in the composition of breast milk protein, we believe that impact of maternal diet on the amino acid pattern in breast milk requires further research to assure the optimal nutrition for breastfed infants^(31,32)^.

Foods with high protein quality (DIAAS ≥ 100) are important to bridge the nutritional gaps created by consumption of the low-quality plant-based staple foods. Animal protein sources such as dairy products, beef, chicken and eggs were animal-source foods shown to be such excellent protein sources (DIAAS ≥ 100; Table 10)^(41–43)^. These foods could be of importance in bridging the gap between the lack of lysine from cereals and legumes. Our results showed that about 40 % of the Nigerian population eats beef frequently. Eggs and chicken are eaten frequently by one-quarter of the Nigerian population. Milk was frequently consumed by one-third of the population. It was shown that plant-based foods had an average DIAAS of 61, whereas animal-source foods had an average DIAAS of 114^(41)^. If these animal-source foods are consumed daily, they could replenish the amino acids that are consumed in inadequate amounts due to the daily consumption of mainly cereals. A relatively high-quality plant-based food is soya beans which was shown to be an excellent source of protein and could, therefore, be a valuable (plant food) addition to the Nigerian diet (Table 10)^(41)^.

**Table 10.** Digestible indispensable amino acid score (DIAAS) of high-quality protein foods (adapted from Ertl *et al.* ^(41)^)

Food	DIAAS (limiting amino acid)*
Beef	112
Milk	116 118 (SAA)† 120 (SAA)‡ 141 (SAA)§
Chicken	108
Eggs	116
Soya beans	100

SAA, sulfur amino acids (methionine and cysteine).

* Based on the scoring pattern of 6 months–3 years using pig true ileal digestibility factors.

† Adapted from Rutherfurd *et al.* ^(43)^ based on the scoring pattern of 6 months–3 years using rat true ileal digestibility factors.

‡ Adapted from Mathai *et al.* ^(42)^ based on the scoring pattern of 6 months–3 years using pig true ileal digestibility factors.

§ Adapted from Mathai *et al.* ^(42)^ based on a scoring pattern of >3 years of age using pig true ileal digestibility factors.

### Dietary diversity and protein quality of a food mixture

As a rule, it applies that eating from multiple food groups, being both plant and animal foods, increases the chance of meeting the nutrient requirements^(34,44)^. Dietary diversity is widely recognised as a key component of high-quality diets, as consuming a variety of foods across and within different food groups helps ensure adequate intake levels of essential nutrients^(45)^. However, previous literature concluded that dietary diversity was low in all six examined states in Nigeria and should be increased^(35)^. Resource-poor settings are generally characterised by consumption of monotonous diets^(46)^.

Consumption from animal-source food groups was found to be significantly related to nutrient adequacy in Nigeria^(23)^. Animal-source foods are still regarded as the best source of good-quality protein, since plant proteins, with the exception of soya, are lacking one or more essential amino acids^(41,47)^. Plant foods can make a valuable contribution to overall protein intake, with cereals such as rice and wheat providing adequate amounts of all essential amino acids apart from lysine. The addition of small amounts of animal-based proteins such as milk, cheese, eggs and meat to the diet can possibly bridge the lysine gap that legumes (apart from soya beans) and cereals leave behind.

To demonstrate this in the present review we examined an example of a mixture of commonly eaten plant- and animal-based foods: rice, cowpeas and tilapia (Table 11). The combination of rice, beans and fish is frequently eaten in Nigeria. Portion sizes for rice (51·9 g) and beans (cowpeas) (54·9 g) were obtained from a study in southwestern Nigeria^(22)^. A portion size of 50 g was assumed for tilapia. The same true ileal digestibility factors and amino acid contents of the foods as for the individual food calculations (Tables 4, 7 and 8) were used. The first limiting amino acids were tryptophan and valine. However, the tryptophan content of cowpeas was not determined. Therefore, the DIAAS based on the second limiting amino acids (isoleucine, lysine and tryptophan) was also determined. If we exclude tryptophan, the DIAAS for infants, children and others were, respectively, 51, 76 and 84 %. For young children and individuals older than 3 years the protein quality for this mixture was good (> 75 %). Where rice and cowpeas individually had lysine as the first limiting amino acid for individuals older than 3 years, this mixture of foods is of good protein quality in terms of lysine (reference ratio = 0·90) for this age group and tilapia bridged the lysine gap that rice and cowpeas left behind. It should still be examined whether the addition of such foods is feasible in terms of availability, affordability and sustainability. Importantly, when a similar exercise was performed by Suri *et al.* in 2014^(48)^, without the addition of an animal protein source, it was shown that both the addition of groundnuts and cowpeas to cereals like maize, millet and sorghum did not yield good protein quality blends (PDCAAS = 42–67 %). The addition of milk (DIAAS > 116) to the diet might be also valuable for increasing linear growth in stunted children^(49)^ and could be easily achieved by adding milk to pap given to young children.

**Table 11.** Digestible indispensable amino acid score (DIAAS) of a mixture of rice, cowpeas and tilapia (mixed meal) using the method of the FAO^(19)^

	Portion (g)*			Protein (g/100 g)†		Protein content in portion‡	
Rice	51·9			6·9		3·6	
Cowpeas (white coat)	54·9			21·4		11·7	
Tilapia	50·0			18·8		9·4	
Total	156·8			47·1		24·7	

Amino acids	True ileal digestible AA content§			Total in mixture mg of each AA in a total protein‖	Reference ratio scoring pattern infants¶	Reference ratio scoring pattern children	Reference ratio scoring pattern other
	Rice	Cowpeas	Tilapia				
Histidine	81·54	196·04	183·77	18·7	0·89	0·93	1·17
Isoleucine	94·00	273·20	323·45	27·9	0·51	0·87	0·93
Leucine	223·35	517·46	615·89	54·9	0·57	0·83	0·90
Lysine	118·61	276·93	673·13	43·2	0·63	0·76	0·90
SAA	174·61	179·10	342·16	28·1	0·85	1·04	1·22
AAA	270·48	624·20	538·34	57·9	0·62	1·11	1·41
Threonine	99·84	199·09	383·99	27·6	0·63	0·89	1·10
Tryptophan	25·50	n.d.	119·76	5·9	0·35	0·69	0·89
Valine	179·63	271·25	380·70	33·6	0·61	0·78	0·84
DIAAS for the mixture (%) first limiting amino acid**					35 (Tryptophan)	69 (Tryptophan)	84 (Valine)
DIAAS for the mixture (%) second limiting amino acid					51 (Isoleucine)	76 (Lysine)	89 (Tryptophan)

AA, amino acid; SAA, sulfur amino acids (methionine and cysteine); AAA, aromatic amino acids (phenylalanine and tyrosine); n.d., not determined.

* Portion sizes (single serving) for rice and cowpeas were adapted from Sanusi & Olurin^(22)^ and the portion size of tilapia was set at 50·0 g.

† Protein content of raw foods adapted from Stadlmayr *et al.* ^(82)^.

‡ Calculated as portion (g) × protein (g/100 g)/100.

§ Calculated as (amino acid content of foods (mg/g protein) × true ileal digestibility factors of amino acids in the same food) × (protein content in portion per food). The amino acid contents of rice and fish were adapted from Shaheen *et al.* ^(71)^ and for cowpeas from Olaleke *et al.* ^(91)^. The true ileal digestibility factors were adapted from Gilani *et al.* ^(85)^.

║ Calculated as the sum of each amino acid content per food/total protein content in the mixture (24·7 g).

¶ Calculated as total in mixture mg of each amino acid in total protein/scoring patterns. Scoring patterns for infants (birth–6 months), children aged 6 months to 3 years and other individuals older than 3 years were adapted from FAO^(19)^.

** Digestible indispensable amino acid score (DIAAS) determined by multiplying the reference ratio of the first (marked red) and second (marked yellow) limiting amino acid by 100 %.

### Strengths

Nigeria is one of the major countries in the West African region and has a complex and diverse food culture; therefore the results of the present review on Nigeria may be also applicable to other countries in West Africa^(11)^. The methodology of this review can be applied to other low- and middle-income countries since the diets are often, just like the Nigerian diet, mostly plant-based (for example, due to costs) and lack variety, which might influence protein intake adequacy. Diet pattern and protein intake as assessed in the present review reflect recent estimates from the year 2000 and onwards. This review considered both the quantity of protein intake and the protein quality of some major staple foods of the Nigerian diet and a mixture of these foods. Moreover, our review used DIAAS, which is the most recent measure for protein quality and can be considered superior to the old measure (PDCAAS). This was, to our knowledge, the first review that calculated DIAAS of foods such as yam and cassava.

### Limitations

The lack of information in the literature on amino acid contents of foods and on true ileal digestibility factors for calculating DIAAS was a major limitation for the present review. Many true ileal digestibility factors are determined in animals, such as pigs, and it is still unsure if these results and methods are also applicable to humans^(21)^. Many studies did not measure the tryptophan content of foods and we could therefore not calculate a score for this essential amino acid. Given the above, DIAAS was calculated only for nine Nigerian foods, which does not reflect the total diet. The measure for protein quality, DIAAS, does not take quantity into account since it calculates protein quality based on the amounts of amino acid (mg) per 1 g of food protein^(19)^. Furthermore, our review did not take anti-nutritional factors, such as trypsin inhibitors (legumes), tannins (legumes and cereals) and phytates (cereals), both present naturally in the food or formed during processing, into account that may possibly interfere with the absorption of nutrients from the diet. The FAO^(19)^ reported that the methods for determining true ileal digestibility factors might not always fully account for anti-nutritional factors.

### Recommendations and future research

We calculated protein quality for a mixture of foods, but information on protein quality of more foods should be determined to calculate DIAAS for complete meals (for example, soups, stews with vegetables, etc.), which play an important role in the Nigerian diet. Also, it is advised to determine the protein quality for more mixtures of foods to examine the role of animal-source proteins in bridging the essential amino acid gaps that plant-based foods leave. To do this, more research should be conducted on amino acid contents of such foods and true ileal digestibility factors to broaden the DIAAS calculations. Also, research should examine whether animal-source proteins can increase the dietary diversity score of Nigerian individuals. Related to this, future research should aim at studying the effects of dietary diversity on protein and amino acid adequacy. The distinction between protein quality *v.* quantity should be made clearer. This might help in determining portion sizes of (mixtures) of foods for meeting the essential amino acid requirements.

Another attention point for future research might be the development of specific products that address the protein and amino acid needs for those individuals that currently do not consume enough protein. For example, for adolescent girls, it may be beneficial to enrich often consumed snacks with good-quality proteins from, for example, soyabean flour^(50)^ or dairy products. Also, possibilities of enriching complementary foods for children, like pap, with good-quality proteins such as soya beans, fish or milk could be explored further^(51,52)^. Providing children with a good-quality protein-rich lunch that will account for about one-third of the protein recommendations in the context of a school feeding programme, for example, might help in eradicating PEM^(53,54)^.

Previous Nigerian studies have suggested affordable and available protein-rich foods such as rabbit meat^(55)^, insects^(56)^, wild plant species^(57)^, indigenous leafy vegetables^(12)^ and goat milk^(58)^. Some studies have already been conducted on determinants (for example, socio-economic factors, education, infections) of protein intake and PEM, and it is advised to extend this knowledge to better understand the context of protein malnutrition^(4,59,60)^. Also, we believe further research into the effects of maternal diets deficient in essential amino acids on the breast milk amino acid composition is warranted. Finally, we suggest applying the methodology of this review to other developing countries to examine if there is a trend in protein intake adequacy across these countries.

## Conclusions

Overall, it was shown that Nigerian population groups had adequate protein intake. The troubling exceptions are adolescent girls and pregnant and lactating women. Furthermore, the Nigerian diet consists mainly of cereals and other plant-based foods, with animal-source foods being consumed to a lesser extent. These results might also be expected in other developing countries since animal-source foods are often more expensive. The protein quality of all plant-based foods as assessed in the present review was shown to be poor when looking at the first limiting amino acids. Rice and wheat were shown to be good sources of protein when looking at the second limiting amino acids. The most limiting amino acids were lysine, leucine, valine, SAA and isoleucine. Tilapia was shown to be an excellent source of protein for individuals above 3 years of age. A mixture of foods from different food groups (rice, cowpeas and tilapia) was shown to be of good protein quality (DIAAS > 75). The addition of animal-source foods bridged the protein quality gap that was created by a predominance of plant-based foods in the Nigerian diet. For example, milk, with its excellent protein quality, could be a valuable addition to the Nigerian diet, especially for malnourished children. Future steps include determination of protein quality of more foods and mixtures and developing and promoting good-quality protein-rich products or meals, which are cost-effective.
